# Honeycomb-Shaped Phononic Crystals on 42°Y-X LiTaO_3_/SiO_2_/Poly-Si/Si Substrate for Improved Performance and Miniaturization

**DOI:** 10.3390/mi15101256

**Published:** 2024-10-14

**Authors:** Panliang Tang, Hongzhi Pan, Temesgen Bailie Workie, Jia Mi, Jingfu Bao, Ken-ya Hashimoto

**Affiliations:** 1 School of Integrated Circuit Science and Engineering, University of Electronic Science and Technology of China, No. 2006, Xiyuan Avenu, West Hi-Tech Zone, Chengdu 611731, China; mailfortpl@163.com (P.T.); wtbailie@std.uestc.edu.cn (T.B.W.); k.hashimoto@ieee.org (K.-y.H.); 2 CETC No. 26 Research Institute, 14#Huayuan Road, Nanping, Nanan District, Chongqing 400060, China

**Keywords:** multi-layered structure, phononic crystals, conventional SAW resonator, reflective grating

## Abstract

A SAW device with a multi-layered piezoelectric substrate has excellent performance due to its high Q value. A multi-layer piezoelectric substrate combined with phononic crystal structures capable of acoustic wave reflection with a very small array can achieve miniaturization and high performance. In this paper, a honeycomb-shaped phononic crystal structure based on 42°Y-X LT/SiO_2_/poly-Si/Si-layered substrate is proposed. The analysis of the bandgap distribution under various filling fractions was carried out using dispersion and transmission characteristics. In order to study the application of PnCs in SAW devices, one-port resonators with different reflectors were compared and analyzed. Based on the frequency response curves and Bode-Q value curves, it was found that when the HC-PnC structure is used as a reflector, it can not only improve the transmission loss of the resonator but also reduce the size of the device.

## 1. Introduction

The accelerating pace of life and the constraints of space have led to a growing demand for portable and easily carried products. Smartphones and wearable devices have made it possible for people to enjoy convenience anytime and anywhere, and have become indispensable in people’s lives. Currently, the design and manufacturing technologies have been significantly improved. Under the premise of ensuring functionality and quality, the market’s pursuit of a smaller size and lighter weight is intensifying, further propelling the trend in electronic product miniaturization.

In recent years, significant advancements in surface acoustic wave (SAW) design and manufacturing technology have given it obvious advantages over other competing technologies, including compact size, excellent performance, high stability, low cost, and simple fabrication processes. These advantages have made it a key component in various radio frequency (RF) and microwave systems, such as radar, wireless communication, satellite navigation, and sensing applications [1,2,3,4,5].

Traditionally, SAW filters are fabricated on Lithium Niobate (LN) or Lithium Tantalate (LT) bulk substrates [6], benefit from the highly developed processing technology of bulk wafers, and their price is extremely affordable. However, due to the limitations of these conventional bulk wafers’ material properties, it is difficult for them to appear in the high-performance filter market [7]. Researchers have made great efforts in improving the performances of substrates in recent years. As an advanced SAW filter technology, the incredibly high-performance (IHP) SAW has great competitiveness and broad application prospects. A SAW filter with this multi-layered substrate exhibits an extremely high quality-factor (Q) value, even exceeding 3000 [7,8,9,10,11,12], which is superior to the traditional SAW filter’s 1000. The low-temperature coefficient of frequency (TCF) enables it to maintain a stable frequency response at various ambient temperatures combined with a significant increase in power capacity, providing more reliable performance for wireless communication systems.

The structure of a standard SAW resonator consists of an interdigital transducer (IDT) and two reflectors (ref) placed on either side of the acoustic channel. Typically, the number of metal strips in each reflector is between 10 and 50, whereas the number of IDT fingers is between 80 and 350. Generally, more resonators imply that the system can handle more complex frequency responses, and larger resonators may exhibit higher Q. When the filters need a sharp cut-off characteristic and high out-of-band rejection, the number and size of resonators exhibit a positively correlated relationship with performance to a certain extent. In some cases, due to the absence of a displacement component perpendicular to the substrate surface in shear horizontal (SH) waves, the reflection efficiency of traditional finger electrodes is notably decreased [13,14]. As a result, it becomes necessary to use a much larger number of electrodes to excite the wave, which leads to an increased device area. Therefore, traditional SAW devices cannot simultaneously balance performance and size, and miniaturization has become a very important research topic.

Advancements in micro-nano technology and photolithography have significantly decreased the size of SAW filters. The implementation of new materials with low velocity also has provided high performance and the potential for further miniaturization. In addition to the above, by optimizing the structure of the filter, such as adding phononic crystals (PnCs), the size of the filter can be reduced while ensuring performance [15,16,17].

Phononic crystals provide bandgap through which an acoustic wave with frequency in the bandgap range cannot propagate within the structure formed by periodic array unit cells [18,19,20,21,22,23]. Previous studies, such as [24,25,26,27], had already employed PnCs on tethers to minimize anchor loss. The authors of [28] achieved superior Q values by replacing metal strips in a SAW resonator with PnC on a bulk substrate. The widespread application of IHP SAW will also locate miniaturization as one of its key development objectives. In this regard, combining the advantages of PnC and multi-layered substrate is a very important way and research direction for SAW to realize high-performance filters while achieving miniaturization. 

In this paper, a phononic crystal structure was designed based on 42°Y–X LT/SiO_2_/poly-Si/Si multi-layered substrate. We conducted a series of comprehensive analyses of the surface acoustic wave model with PnC using analytical theory and a finite element method. Firstly, we studied the constitutive relationship of phonon vibration and then analyzed the dispersion characteristics of the bandgap formed by PnC. Then, the bandgap distribution and displacement transmission characteristics under a different filling fraction (FF) were analyzed by the finite element method. Based on the abovementioned analysis, the appropriate structural configuration of the multi-layer substrate-matched PnC was obtained. A comparative analysis was undertaken to evaluate the performance of resonators by adding PnC or metal grating as reflectors with identical dimensions. Moreover, in order to ensure the influence of etching depth on PnC, a one-port resonator with a different PnC thickness was analyzed.

## 2. Phononic Crystal Design

When acoustic waves propagate in an elastic medium, the force acting on each face of an infinitesimal volume element can be decomposed into one component perpendicular to the surface and two components parallel to the surface. Using Newton’s Second Law of Motion and Einstein’s summation convention, the motion equation for the elastic medium within the aforementioned stress coordinate system can be expressed as follows:

(1) $$\frac{\partial T_{ij}}{\partial x_{j}}=\rho \frac{\partial^{2}u_{i}}{\partial t^{2}}\;\;(i,\;j\;=\;1,\;2,\;3)$$

where $T_{1j}$, $T_{2j},$ and $T_{3j}$ are stresses in three directions parallel to and perpendicular to the $x_{j}$ axis, respectively; ρ is the density of the elastic medium; $u_{i}$ is the displacement of the medium in the $x_{i}$ direction; and t is the time. According to the generalized Hooke’s law:

(2) $$T_{ij}=c_{ijkl}S_{kl}\;(i,\;j,\;k,\;l\;=\;1,\;2,\;3)$$

where $S_{kl}$ is the strain tensors, and $c_{ijkl}$ is the elastic stiffness constant of the elastic medium. The following relationship exists between strain $S_{kl}$ and displacement $u_{k}$:

(3) $$S_{kl}=\frac{\partial u_{k}}{\partial x_{l}}\;\;(k,\;l\;=\;1,\;2,\;3)$$

Substituting Equation (3) into Equation (1), we obtain the following:

(4) $$\rho \frac{\partial^{2}u_{i}}{\partial t^{2}}=c_{ijkl}\frac{\partial^{2}u_{k}}{\partial x_{l}\partial x_{j}}\;(i,\;j,\;k,\;l\;=\;1,\;2,\;3)$$

Scatterers are periodically distributed along the lattice, and the Bloch–Floquent theorem explains the spatial symmetry of crystals and the translational repeatability of their lattices. Therefore, $u_{i}$ also satisfies the following periodic boundary conditions in PnC:

(5) $$u\left(x+a\right)=e^{-iKa}u\left(x\right)$$

where *a* is the lattice constant with components of $a_{i}$ in the $x_{i}$ direction. K is the wave vector.

The bandgap is one of the significant acoustic properties of phononic crystals, arising from the interplay of the Bragg scattering principle and the local resonance mechanism. A single phononic crystal may simultaneously exhibit bandgaps formed by different mechanisms. The Bragg scattering mechanism highlights the effect of the periodic structure of PnC on waves. In contrast, the local resonance mechanism emphasizes the resonant characteristics of miniaturized scatterers and the intricate interactions of the elastic waves. The Bragg scattering condition is as follows:

(6) 2a=nλ

Due to $K=\frac{2\pi }{\lambda }$, Equation (6) can be expressed as

(7) $$K=n\frac{\pi }{a}$$

Thus, acoustic waves satisfying this condition can achieve Bragg reflection within the phononic crystal. As the wave vector *k* changes, the intrinsic motion states and the eigenfrequency of the lattice also change correspondingly. The relationship between the wave vector and the eigenfrequency is also called the bandgap structure diagram. Through researching at the edge of the Brillouin zone, the planar wave dispersion relation and bandgap of the PnC can be found.

**Figure 1:**
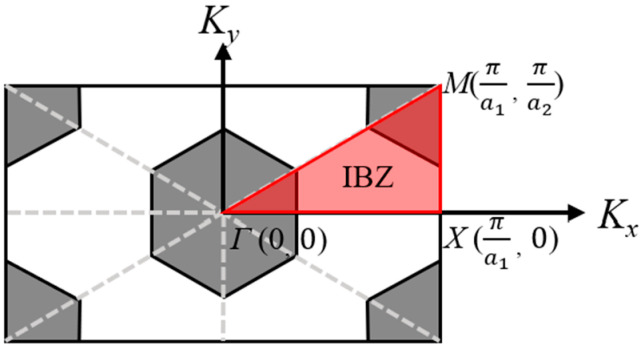
Top view of HC-PnC unit cell with its IBZ.

**Figure 2:**
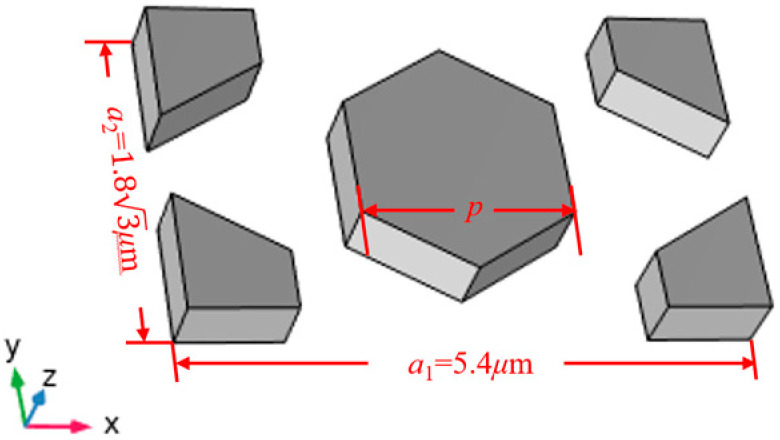
Three-dimensional view of the HC-PnC unit cell.

As shown in Figure 1 of the first irreducible Brillouin zone (IBZ) in the XY coordinate system, PnCs are regular hexagons and exist in a honeycomb shape. Figure 2 shows the first irreducible Brillouin zone in the XYZ coordinate system, calculating the eigenfrequency of the model by a swept finite element simulation. When k changes from 0 to 1, 1 to 2, and 2 to 3, it indicates that the wave vector K sweeps from point Γ to X, X to M, and M to Γ in the first Brillouin zone. The values of the corresponding components $K_{x}$ and $K_{y}$ of the wave vector K are as follows:

(8) $$K_{x}=\left\{\begin{array}{cc} \frac{\pi }{a1}\cdot k & 0<k<1\\ \frac{\pi }{a1} & 1<k<2\\ \frac{\left(3-k\right)\cdot \pi }{a1} & 2<k<3 \end{array}\right.$$

(9) $$K_{y}=\left\{\begin{array}{cc} 0 & 0<k<1\\ \frac{\left(k-1\right)\cdot \pi }{a2} & 1<k<2\\ \frac{\left(3-k\right)\cdot \pi }{a2} & 2<k<3 \end{array}\right.$$

The bandgap width and reflection intensity of PnCs are related to the material, structure, and filling fraction. In order to obtain the appropriate distribution and width of the bandgap, a honeycomb-shaped PnC (HC-PnC) structure was designed based on a multi-layered substrate, as shown in Figure 2.

For this study, the phononic crystal structure was designed based on a 42°Y–X LiTaO_3_/SiO_2_/poly-Si/Si multi-layered substrate. 42°Y-X LiTaO_3_ thin film, as the piezoelectric layer, has exceptional piezoelectric properties and high quality-factors. SiO_2_, serving as a dielectric between the substrate and piezoelectric layers, not only effectively reflects acoustic waves but also provides temperature compensation, ensuring the stable performance of the SAW. Polysilicon (poly-Si) material was used as the trapping layer and Si as the support substrate. In this case, the PnC unit cell lattice constant $a_{1}$ is 5.4 μm and $a_{2}$ is 3.12 μm. The LT is fixed to 600 nm, the SiO_2_ thickness is 500 nm, and the poly-Si thickness is 1000 nm.

**Figure 3:**
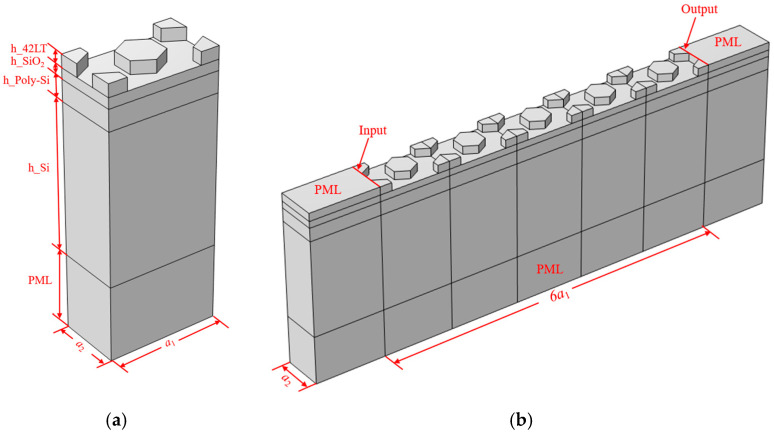
(**a**) Schematic 3D representation of the proposed PnC; (**b**) schematic representation of a transmission line composed of an array of 5 unit cells of the proposed PnC.

**Figure 4:**
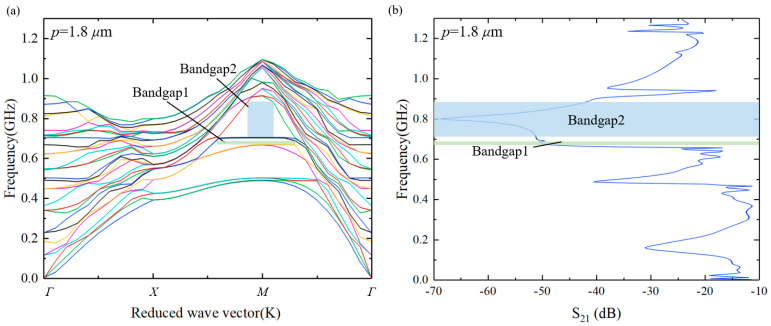
(**a**) Dispersion curve of acoustic waves for the HC-PnC; (**b**) simulated transmission curve through a finite strip.

In order to study the acoustic performance of this HC-PnC, a unit finite element 3D periodic model of the phonon crystal cell was established, as shown in Figure 3a. The material constants used in the calculation are listed in Table 1. A perfectly matching layer (PML) was used at the bottom of the substrate for absorbing the wave propagated into the substrate. The continuity periodic boundaries were set in the X and Y directions, respectively, to obtain the infinite cascading of the PnC unit cells. The eigenfrequency of this infinite periodic model was calculated through a parametric scanning of k. The scanning range was from 0 to 3. Figure 4a presents the dispersion curve of acoustic waves for unit HC-PnC. Different-colored lines represent the different vibration modes. The bandgaps are located within the inflection point of the two lines. At the same time, we simulated the HC-PnC transmission characteristics in the X direction. In order to simplify the solution while maintaining a good enough accuracy, a finite-length strip composed of 5 HC-PnC cells was modeled, as shown in Figure 3b. PML was set on both sides apart from the bottom. The prescribed displacement of 1 pm is used to represent the acoustic vibration at the input point. When an acoustic wave is propagated through PnCs, the transmission response can be obtained by monitoring the mechanical amplitude at the output point.

In Figure 4, the green and blue zones represent bandgap1 and bandgap2, respectively, with the corresponding frequency ranges of 667.6~686.9 MHz and 704~882.4 MHz depicted in both Figure 4a,b. As mentioned, the maximum transmission losses are obtained in these two bandgaps. In addition to the distinct bandgap1 and bandgap2, Figure 4b also exhibits some peaks at the frequency of 485 MHz, 950 MHz, and so on, indicating the presence of bandgaps near these frequency points. However, due to the narrow frequency band range, the transmission loss appears as some sharp peaks in Figure 4b. Using Equation (10):

(10) $$FBG=\frac{f_{u}-f_{l}}{(f_{u}+f_{l})/2}$$

the fractional acoustic bandgap (*FBG*) of bandgap1 and bandgap2 were calculated as 2.8% and 22.5%, respectively, where $f_{u}$ and $f_{l}$ are the upper and lower bounds of the bandgap.

**Figure 5:**
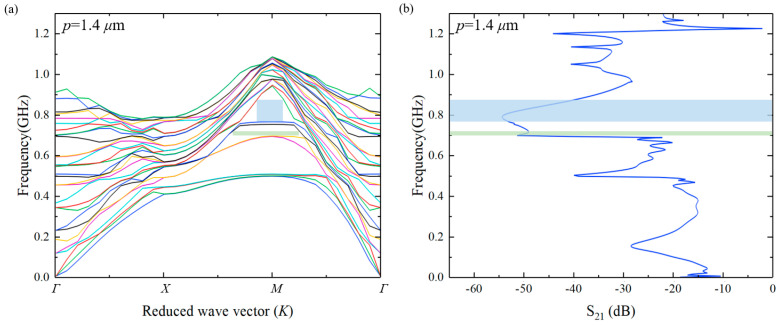

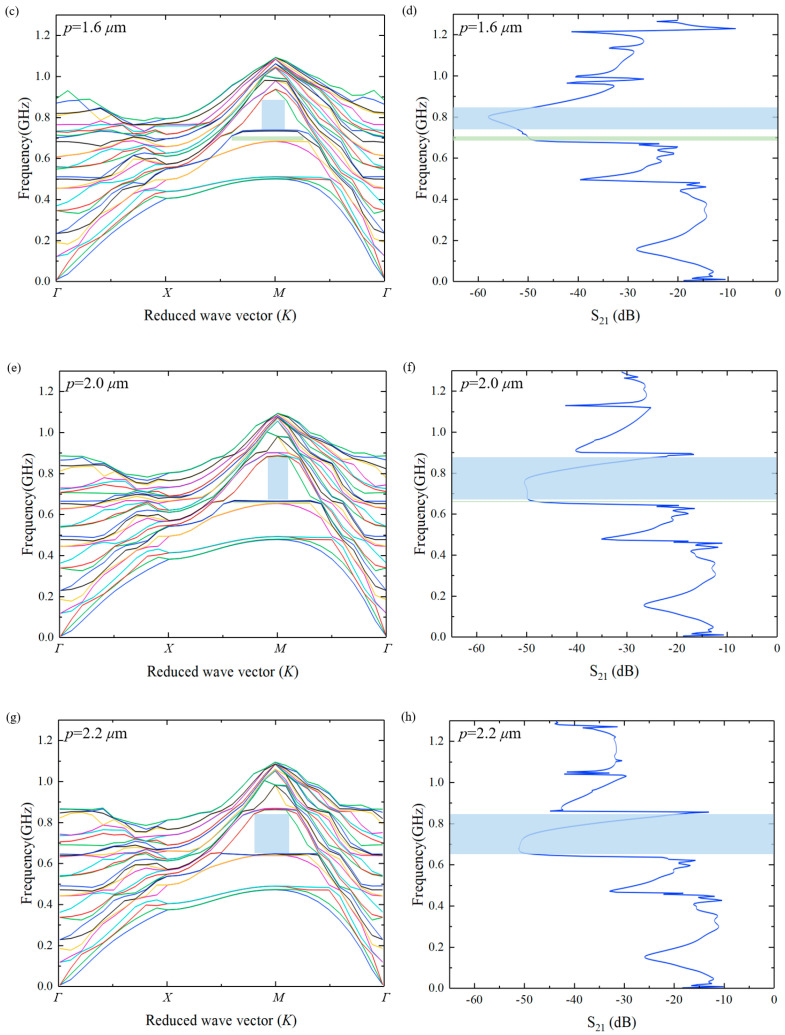
Dispersion curve and simulated transmission curve with different filling fractions. (**a**) dispersion curve with *p* = 1.4 μm. (**b**) simulated transmission curve with *p* = 1.4 μm. (**c**) dispersion curve with *p* = 1.6 μm. (**d**) simulated transmission curve with p = 1.6 μm. (**e**) dispersion curve with *p* = 2.0 μm. (**f**) simulated transmission curve with *p* = 2.0 μm. (**g**) dispersion curve with *p* = 2.2 μm. (**h**) simulated transmission curve with *p* = 2.2 μm.

Due to the use of fixed-thickness IHP wafers in this study, however, the influence of the thickness of each material layer on the PnCs is not considered for the time being. Figure 5 analyzes the distribution of bandgaps under different filling fractions. The filling fraction is the ratio of the hexagonal graphical area to the lattice area:

(11) $$FF=\frac{\sqrt{3}p^{2}}{a_{1}\cdot a_{2}\;}$$

It can be seen that with the increasing filling fraction, the upper and lower edge frequencies of each bandgap decrease, but FBG does not change linearly, as shown in Table 2. It is clear that the FBG of bandgap1 exhibits a nonlinear change with the increasing hexagonal width. The FBG reaches its maximum at around *p* = 1.6 μm and gradually decreases until it disappears. However, with the increasing of *p*, the width of bandgap2, which can obtain the maximum transmission characteristics, gradually decreases. This is due to the obvious change in the lower-edge frequency of bandgap2, resulting in a nonlinear increase in the FBG. The maximum loss of transmission (S21-dB) was obtained at *p* = 1.8 μm within bandgap2. In addition to bandgap1 and bandgap2, S21-dB also exhibits some peaks at other frequencies under various FFs, which are also manifestations of extremely narrow bandgaps.

## 3. Results and Discussion

In conventional SAW devices, metal reflecting gratings on both sides of the IDT acoustic path are used as reflectors to form Bragg reflection to make the resonant frequency within the stopgap. For resonators using PnC as a reflector, if the SAW resonant frequency is within the bandgap range of the PnC, it can also reflect acoustic waves in the IDT region and improve the loss of the SAW resonator.

**Figure 6:**
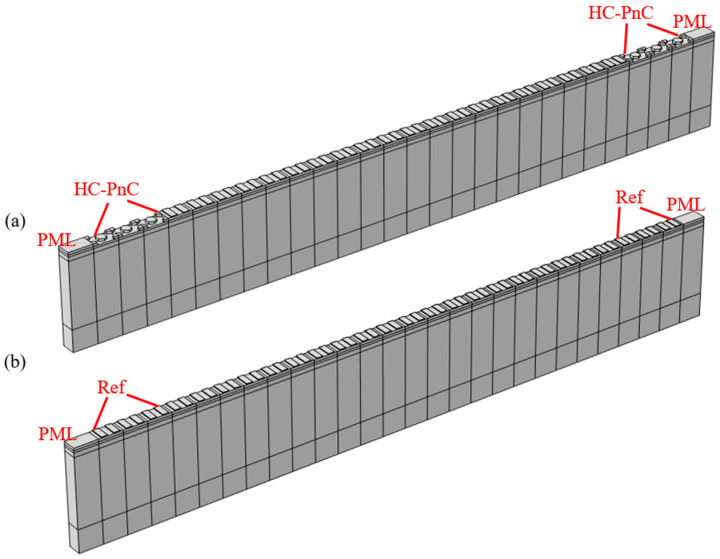
A schematic 3D representation of a one-port SAW resonator with (**a**) 3 HC-PnCs as a reflector and (**b**) 3 pairs of reflective gratings.

As shown in Figure 6a, a one-port SAW resonator 3D model was established, in which the IDT consists of 20 pairs of aluminum electrodes with a thickness of 200 nm. The IDT period λ was 5.4 µm and the metallization ratio of the IDT was 0.5. Three HC-PnC lattices as reflectors were located on either side of the IDT. The aperture length was a2, and the continuity periodic boundary conditions were imposed on the boundaries in the depth direction. Since the energy of surface acoustic waves is mainly concentrated on the surface of the piezoelectric film, the grid size in the area below the electrode was smaller than that in other areas of the substrate. The maximum grid size was λ/6. Through the transmission curve, we obtained a resonant frequency of 750 MHz, which was in bandgap2 with a hexagonal width of *p* = 1.8 μm. A one-port SAW resonator 3D model with three pairs of reflective gratings was also established. The reflective grating period λ was 5.4 µm, too. Therefore, the size of this structure was precisely the same as the previous one.

**Figure 7:**
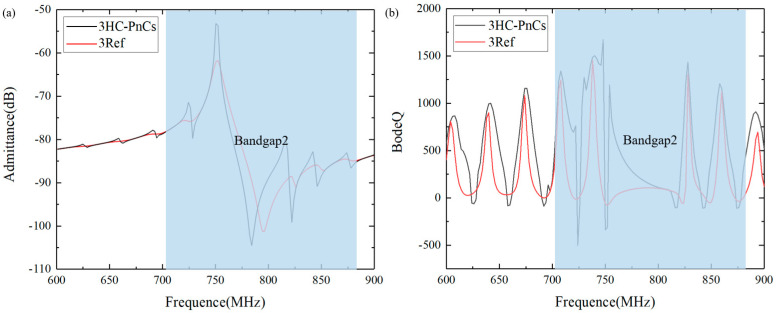
(**a**) Simulated admittance (Y) response and (**b**) simulated Bode-Q of a one-port SAW resonator with 3HC-PnCs as a reflector vs. 3 pairs of reflective gratings.

Figure 7a comparatively analyzed the frequency response of the resonators accompanied with three HC-PnCs and three pairs of reflective gratings. It can be observed that the amplitude of the resonant peak of the PnCs is much greater than that of the reflection gratings. Besides the resonant peak, some spurious waves appeared nearby, all of which exhibited increased amplitudes. This is due to the limited number of electrodes in the IDT, which resulted in weak coupling between the electricity and the acoustic waves and diminished the amplitude difference between the main mode and spurious modes. As the number of fingers in the IDT increases, the main mode’s excitation efficiency will be higher, while the other acoustic wave modes will be weaker. Meanwhile, since these ripples resided within the bandgap, the reflected energy was also intensified.

The Bode-Q serves as a crucial indicator of resonators’ capacity to store energy, and it significantly impacts insert loss, out-of-band rejection, and the cut-off characteristics of SAW devices. Figure 7b presents the Bode-Q results calculated for both cases. As illustrated in the figure, the Q value of the resonator with HC-PnC is 812 at 750 MHz, whereas, for the alternative approach, it is only 71 at the same frequency. Furthermore, it is noteworthy that the lattice width a1 is equal to the reflection grating period. The results indicate that, in comparison to metal reflective gratings, the resonator with HC-PnC exhibited a significant advantage in Q value near the resonance peak. Conversely, to achieve the same Q value as the HC-PnC structure, the metal electrode reflector structure requires much more gratings, which will greatly increase the size of the device.

**Figure 8:**
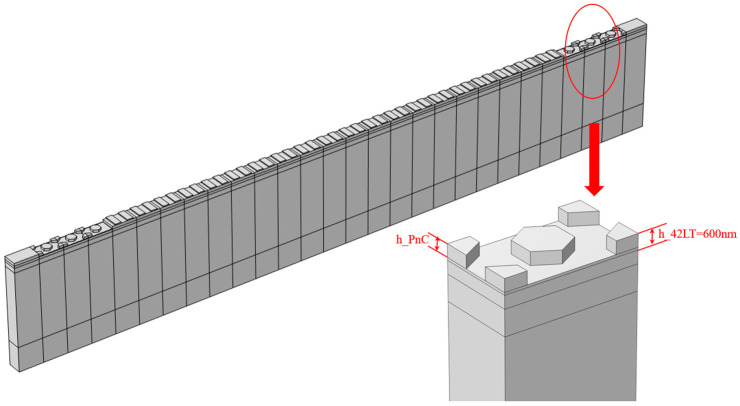
A one-port SAW resonator 3D model with an incomplete etched LT layer and the detailed model.

**Figure 9:**
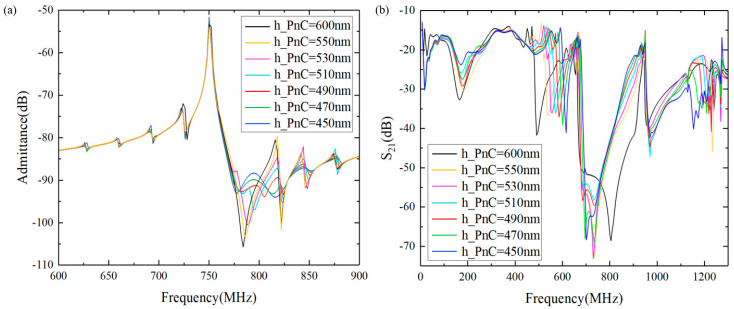
(**a**) Simulated frequency response curve and (**b**) simulated transmission curve with different PnC thicknesses.

Taking into account the actual processing conditions, we have investigated the impact of different LT-etched thicknesses on the bandgap width using the same piezoelectric-on-insulator (POI) substrate. Figure 8 shows a detailed model of this configuration. The frequency–response curves of acoustic–electric coupling and the displacement transmission–response curves of the resonator were calculated for various LT thicknesses, as illustrated in Figure 9. From Figure 9a, it can be observed that when the PnC thickness is 550 nm, there is no significant difference in frequency response compared to 600 nm. However, for PnC thicknesses below 550 nm, the anti-resonance point began to exhibit noticeable changes. Figure 9b demonstrates that as the thickness of the PnC diminished, the bandgap contracted, and the upper-edge frequency of the bandgap decreased, causing the anti-resonance point to drop outside the bandgap or to move into another bandgap. The above simulations indicate that the thickness of the PnC also has a significant impact on the bandgap. Therefore, the thickness of the PnC should be strictly controlled during actual processing.

## 4. Conclusions

In this paper, a honeycomb-shaped phononic crystal based on 42°Y-X LT/SiO_2_/poly-Si/Si-layered substrate was studied. A comprehensive analysis was performed on the proposed HC-PnC 3D finite element model. The dispersion and transmission curves were calculated, which are required to determine if there is good energy confinement in bandgap. It was found that this PnC structure has multiple bandgaps. As the FF increases, the upper and lower bounds of the different bandgaps gradually decrease, but the FBG does not change linearly. Furthermore, 3D models of one-port resonators with two distinct types of reflectors were established using the multi-layered structure of Al/42°Y-X LT/SiO_2_/poly-Si/Si, followed by an analysis of the simulated outcomes. After comparing the results of admittance and the Bode-Q value of the resonator using HC-PnC as a reflector versus reflective gratings as reflectors, it was determined that the HC-PnC structure significantly enhances acoustic reflection. Given the same reflected energy, the resonator with reflective grating is several times larger than the one with HC-PnC. Meanwhile, it is imperative to exercise rigorous control over the thickness of PnC during processing in order to mitigate the impact of bandgap-shifting as analyzed.

The PnC reflector on a POI substrate offers a promising avenue of research for achieving miniaturization and enhancing the performance of SAW devices simultaneously.

## Figures and Tables

**Table 1:** Material constants used in the calculation.

	Symbol	LiTaO_3_	SiO_2_	Poly-Si	Si
Elastic Constants (×10^10^ N/m^2^)	*C*_11_	23.29	7.85	-	-
	*C*_12_	46.89	1.61		
	*C*_13_	80.23	1.61		
	*C*_33_	27.53	7.85		
	*C*_44_	93.89	3.12		
Piezoelectric Constants (C/m^2^)	*e*_15_	2.59	-	-	-
	*e*_31_	0.08			
	*e*_33_	1.88			
Dielectric Constants	$\varepsilon_{11}/\varepsilon_{0}$	40.9	3.75	4.5	11.7
	$\varepsilon_{33}/\varepsilon_{0}$	43.3	3.75		
Density (kg/m^2^)	ρ	7450	2200	2320	2329

**Table 2:** Bandgap and FBG under different filling fractions.

Hexagonal Width *p* [um]	Bandgap1 [MHz] (FBG)	Bandgap2 [MHz] (FBG)	*FF*
1.4	704.0~724 (2.8%)	794.7~883. 6 (10. 6%)	20.1%
1.6	683.1~707.6 (3.5%)	744.4~882.4 (16.9%)	26.3%
1.8	667.6~686.9 (2.8%)	704~882.2 (22. 5%)	33.3%
2.0	652.5~662.7 (2.5%)	675.1~878.8 (26.2%)	41.2%
2.2	-	654.3~859.0 (27.1%)	49.8%

## Data Availability

The original contributions presented in the study are included in the article.
